# Study of the impact of introducing a multimedia learning tool in podiatric medical courses

**DOI:** 10.1002/jfa2.12018

**Published:** 2024-06-29

**Authors:** Garrik Hoyt, Samuel Adegboyega, Gus Constantouris, Paramita Basu

**Affiliations:** ^1^ Touro University New York New York USA; ^2^ New York College of Podiatric Medicine New York New York USA

**Keywords:** audiovisual learning resource, knowledge retention, medical education, mnemonics, multimedia learning tool, podiatric medical education

## Abstract

**Background:**

Medical students face the challenge of learning vast amounts of complex information. Existing research suggests improved learning outcomes using multimedia resources but reports on their impact on podiatric education are scarce. To explore the potential of multimedia‐based learning tools in enriching medical education, this study examined the impact of Osmosis, a platform featuring interactive videos, flashcards, and self‐assessment quizzes on podiatric medical student outcomes.

**Methods:**

This quasi‐experimental study examined the impact of Osmosis, a multimedia learning platform with videos, flashcards, and quizzes, on podiatric medical students' learning outcomes. Two cohorts (*T* = Osmosis access, *N* = 86; *C* = no access, *N* = 87) took Pharmacology and Podiatric Medicine courses consecutively. Final exam scores, final course grades, platform usage metrics (median weekly videos watched, flashcards, and quizzes), and student experience surveys were analyzed.

**Results:**

Analyses revealed no statistically significant differences in final exam scores between the groups in Pharmacology and Podiatric Medicine. While the treatment group exhibited a slight upward trend, further research is required for conclusive evidence. Student perceptions of Osmosis were overwhelmingly positive, with 90.2% of students agreeing that it facilitated concept learning and understanding compared to 54.9% for the textbook. Similarly, 80.4% of the treatment group felt that Osmosis enhanced their test performance, exceeding the 54.9% recorded for the textbook. Correlation analysis indicates a plausible connection between platform usage and academic success, as reflected by moderate positive correlations (*r* = [0.14, 0.28]) with final grades. Logistic regression analysis revealed that students with Osmosis access were 2.88 times more likely to score 90% or higher on the Pharmacology final exam (*p* < 0.05) and exhibited increased odds of achieving high (90%+) final course grades in Podiatric Medicine (OR = 2.71).

**Conclusions:**

These findings suggest that Osmosis holds promise as a tool to support podiatric medical student learning. While the lack of statistically significant differences in final exam scores warrants further investigation, the positive student perceptions, high engagement rates, and increased odds of high scores in specific areas indicate the potential for Osmosis to positively impact academic outcomes. Therefore, a multimedia‐based resource like Osmosis appears to show promise as a tool to support podiatric medical education. The limitations inherent in the quasi‐experimental design necessitate further studies to confirm its effectiveness and long‐term impact on podiatric medical education.

## Graphical abstract

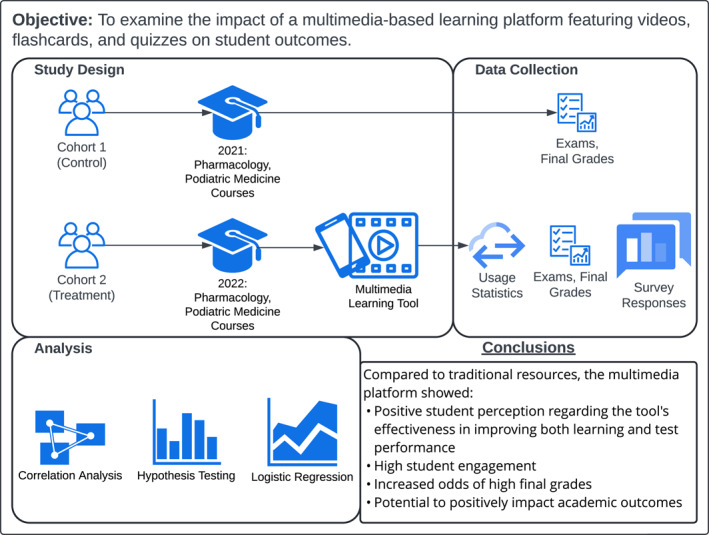

## 1 INTRODUCTION

All medical students, including those pursuing podiatric medicine, face the difficult task of learning immense amounts of information about complex systems and processes. To address this challenge, many medical schools are integrating multimedia learning principles into their curricula to improve the quality of instruction as well as student experience. Institutions have various commercial multimedia learning tools to choose from that incorporate key multimedia learning principles. The cognitive theory of multimedia learning (CTML) revolves around three big ideas, namely the dual channel principle, the limited capacity principle, and the active processing principle. These ideas inform how multimedia learning may improve learning outcomes [1, 2]. Effective multimedia learning resources leverage these principles to facilitate learning and improve student experience using structured digital materials that serve as a learning objective [3]. However, the successful use of electronic technology and media to deliver learning materials and content through online platforms depends on the implementation [4].

In recent years, there have been an increasing number of tools tailored specifically for medical education that incorporate video, animation, and mnemonics [5]. Several quantitative studies have examined the impact of multimedia learning resources on learning outcomes, which were shown to improve learning retention for third year medical students [6], while another meta‐analytical study concluded that animated graphics had a positive effect on knowledge acquisition over static graphics [7]. Additionally, story‐based audiovisual mnemonics have been shown to have a significant impact on test scores as well as time to recall when compared to traditional text reading [8].

Qualitative studies have investigated student sentiment and student experience around multimedia learning tools. Researchers found that medical students want more videos used in training for surgical procedures and that students find video learning useful and easy to use [9, 10]. Key factors to consider in exploring the influence of a multimedia‐based resource on learning outcomes include participation, modification of attitudes and perceptions, modification of knowledge and skills, and change in behavior [3]. In this light, our study aims to investigate the impact of implementing Osmosis (https://www.osmosis.org), a multimedia learning tool that includes videos using audiovisual picture mnemonics, flashcards, clinical decision‐making trees, and concept‐focused notes with illustrations and visual cues for medical students taking preclinical and early clinical courses along with embedded case‐based multiple‐choice quiz questions for self‐assessment of learning. This was achieved with the use of a playlist of selected videos with the accompanying quizzes and flashcards from Osmosis in the podiatric medicine and pharmacology courses to complement the course materials. To analyze the impact, we have explored both qualitative and quantitative measures, including students' usage of the videos, practice quiz questions, and flashcards as part of the multimedia resource, their opinion regarding the platform and its features, comparing student opinions on multimedia learning versus using the course textbook, and its effect on improvement of final exam scores and total course grades in a quasi‐experiment.

There is a dearth of literature on the effectiveness of incorporating multimedia‐based learning tools in Podiatric medicine curriculum, we have tried to address this gap by investigating the potential of using commercially available multimedia resources along with traditional instructional methods for providing spaced repetition to achieve higher learning retention and course performance in Podiatric medicine students. Despite the limitations imposed by its quasi‐experimental design, this study provides valuable insights into the potential impact of multimedia learning on Podiatric medical education.

## 2 METHODS

### 2.1 Study design

This study was approved by New York College of Podiatric Medicine's (NYCPM) Institutional Review Board (IRB) in May 2022. Informed consent was waived off by the IRB since students agree to the use of unidentifiable education data for research purposes at registration. A mixed methods approach was utilized to examine both qualitative and quantitative measures related to student experience and the impact of multimedia learning on medical education.

Two cohorts totaling 173 students participated in this study, comprising second year medical students in the fourth semester at the New York College of Podiatric Medicine. Students belonging to the two cohorts included in the study took the same two courses—Pharmacology and Podiatric Medicine—in consecutive years. Both courses are supported by assigned readings from standard textbooks and instructor‐uploaded content delivered didactically, along with traditional summative assessments in the form of exams. The cohorts consisted of a control (C) group (which is the graduating class of 2024) of 67 students taking the course in 2021 and a treatment (T) group (which is the graduating class of 2025) of 91 students taking the course in 2022. Participants in only the treatment (T) group were provided access to the videos within Osmosis platform and used the multimedia web‐based learning tool as a learning resource to complement the traditional course material, while participants in the control (C) group had access to the traditional course materials but did not have access to the Osmosis videos. All students in both cohorts were given the same didactic instruction, textbooks, and other traditional learning resources. In the treatment group, playlists of assigned videos from Osmosis were recommended to be watched before the live sessions throughout the course (Appendix S1) along with multiple attempts to watch the videos, answer the embedded quiz questions or flashcards. The study was conducted as a posttest‐only nonequivalent group, with a quasi‐experimental design [11, 12]. Although sample selection was non‐random, it is assumed that the two sample groups are similar in their curricular level within the program with similar average standardized test scores, similar mean incoming cumulative GPAs, and were given similar course content and assessments. Other confounders such as educator's quality and digitalization were addressed by using the same instructors and Learning Management System (LMS) for delivery of course content to both the cohorts. The treatment group (T) used Osmosis throughout the second half of the semester; the control group (C) took the same courses without access to Osmosis. Osmosis is a digital education platform that includes videos, flashcards, and case‐based multiple‐choice questions. Students were advised to watch specific videos from playlists compiled by instructors and answer the associated questions and flashcards for review and reinforcement of the assigned topics, after the content was covered in the course. Data on the number of videos watched, number of video‐embedded questions answered, and the number of flashcards answered by the students were monitored and recorded using the platform's administrator dashboard. In the treatment cohort (T group), completion of the assigned Osmosis‐based questions and flashcard accounted for 2% of the total course grade in the form of participation credit. In the Control cohort (C group), the students were given 2% participation credit for active participation in the in‐person class discussions based on prior review of the posted instructional materials, learning resources, and the textbook chapters before live sessions. The contribution of all other course assessments was weighed identically in both control and treatment cohorts. The students' engagement on a weekly basis was monitored by course faculty using Osmosis dashboards that showed video‐viewing data and completion of associated flashcards and questions (Appendix S2).

Final exam scores between the two cohorts were compared using a posttest only nonequivalent group quasi‐experimental design [11]. Cumulative final exam scores were observed for both cohorts in the two courses. In addition, students in the treatment group were emailed an electronic survey after the end of the semester but before the release of final grades, to obtain feedback about student preference and experience about usage of the online multimedia platform. Students were asked to indicate their extent of agreement of whether the textbook helped them to retain information, learn new concepts, and achieve higher test scores prior to accessing Osmosis. Then students were asked to indicate their extent of agreement of whether using Osmosis (in addition to traditional learning resources) helped them to retain information, learn new concepts, and achieve higher test scores to compare students' perception of the usefulness of Osmosis as a learning resource to that of the textbook. Students were also asked to respond to an open‐ended question about which specific features of Osmosis they felt were the most helpful and to rate their satisfaction level for each learning resource (for a traditional resource such as textbook and a multimedia resource such as Osmosis) on a scale from 1 to 5 to compare the students' learning experience with and without access to Osmosis. Platform usage metrics for the treatment group were also gathered at the end of the semester (number of videos watched, flashcards answered, and questions answered) for further analysis to see if there was any correlation between platform usage metrics such as frequency and type of feature usage and course performance or grades.

### 2.2 Data collection and analysis

The incoming GPA for students enrolled in both cohorts were imported from the student information system and the mean incoming GPA were calculated for both the C group and the T group to identify any significant inherent difference in the cohorts due to the nonrandom sample selection before the start of the study. The two cohorts compared in our study, the C and T groups were similar in terms of their curricular level within the program (fourth semester, second year), with similar average incoming standardized test scores at the time of admission to the program and were determined to have similar mean incoming cumulative GPAs at the start of the fourth semester.

An email with a link to an electronic web‐based semi‐structured survey questionnaire consisting of 12 required questions (Appendix S3) was sent to the students in the T group, which they voluntarily filled out. The first four questions (Baseline Questions) numbered 1–4 in the survey instrument were designed to determine compliance with informed consent of participants, enrollment in the targeted courses and to ensure that all participating students were comfortable with digital resources with adequate access to Internet and smart devices to remove any possibility of these factors affecting their course performance. The baseline data collected from these questions were used for standardization purposes only and are therefore not shown. The next four questions (Pre‐Osmosis Questions) numbered 5–8 in the survey instrument, were designed to collect data about the students' learning experience prior to receiving access to the Osmosis digital learning platform. Two of these questions were focused on the textbook as learning resource, while the other two were open‐ended questions designed to collect data on the other resources used by students to learn the course materials before using Osmosis. The last four questions (Post‐Osmosis Questions) numbered 9–12 in the survey questionnaire, were designed to collect data about the students' learning experience after receiving access to the Osmosis digital learning platform and the specific features within this multimedia learning tool that helped them learn the course material. The anonymous survey responses were collected in Microsoft Forms and exported to Excel. The college IRB granted ethical approval for this study. User interaction data from the platform were provided by Osmosis through the platform administrator dashboard. Standard strategies appropriate for this type of quasi‐experimental study design, consisting of non‐randomized sampling and posttest‐only nonequivalent groups, were used for the analysis of the data collected [13].

Data analysis was performed using Excel version 2403 and R Studio 2023.06.2, which is an open‐source platform for data analysis. Exploratory data analysis was conducted in R Studio, incorporating the examination of histograms, boxplots, and Quantile–Quantile plots. Average GPA for the two cohorts were compared using descriptive statistics and a two‐sample *t*‐test. These graphical methodologies were employed to acquire insights into the distribution, central tendencies, and adherence to theoretical distributions. Final exam scores for each course were compared between the two groups and analyzed using two sample *t*‐tests in R Studio. Descriptive statistics were calculated for survey questions. Likert scale responses were analyzed to determine student agreement levels. Open answer responses regarding students' most used features were manually categorized in Excel. Correlation analysis was performed in R Studio to explore potential linear relationships between usage metrics and a student's average final grade over the two courses. Logistic regression analysis was performed to explore the relationship between access to the resource and the odds of a student scoring 90% or higher in the final exam for each course.

## 3 RESULTS

### 3.1 Comparison of student performance among the treatment and control groups

**FIGURE 1:**
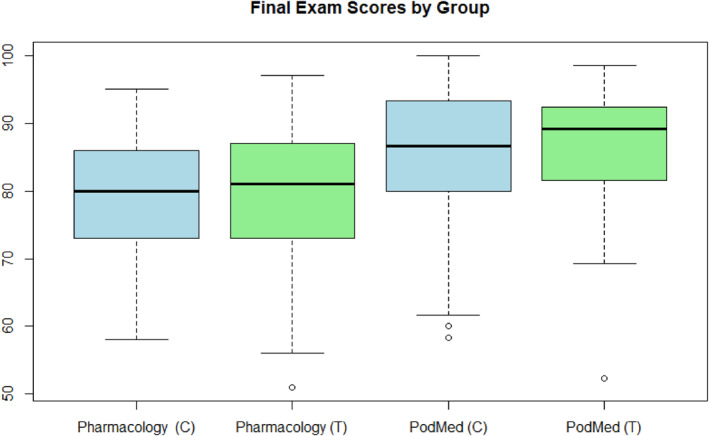
Comparison and distribution of final exam scores obtained by the Control (C) and Treatment (T) groups in Pharmacology and Podiatric Medicine (PodMed) courses. A comparison of student performance in course assessment was made between Treatment (T) group (graduating class of 2025) with access to Osmosis, and Control (C) group (graduating class of 2024) without access to Osmosis, based on their respective Pharmacology and Podiatric Medicine Scaled Mean test scores and Final course grades as well as the Pharmacology, and Podiatric Medicine Score distributions within each group.

We compared student GPAs to determine whether the Control (C) and Treatment (T) groups were similar academically. The control group's mean incoming GPA was 3.04 compared to the treatment group's average of 3.09 (Figure 1). A Welch's two sample *t*‐test indicated that the two groups' incoming GPAs were comparable (*t*(164) = 0.70, *p* = 0.48).

The treatment group scored higher than the control group on the final exams for both courses. The difference in means between the two groups was 1.079 in Pharmacology (*p* = 0.441) and 0.848 in Podiatric Medicine I (*p* = 0.541). Welch's two sample *t*‐tests comparing average final exam scores between the groups indicated no statistically significant difference.

### 3.2 Analysis of students' learning experience survey data

The responses received from the students enrolled in the Treatment (T) group to the four pre‐Osmosis survey questions (numbered 5–8) were analyzed to collect data about the students' learning experience prior to receiving access to the Osmosis digital learning platform. Two of these questions were focused on the usefulness and ease of use of the textbook as a learning resource, while the other two were open‐ended questions designed to enumerate the resources other than the textbooks that were used by students during self‐study, to learn, understand, and retain the course content and information before having access to Osmosis.

The responses received from the Treatment (T) cohort on the post‐Osmosis survey questions (numbered 9–12), were analyzed to collect data about the students' learning experience, perception of usefulness of Osmosis as a learning tool, user satisfaction level, and the preferred usage features after receiving access to the Osmosis digital learning platform.

**FIGURE 2:**
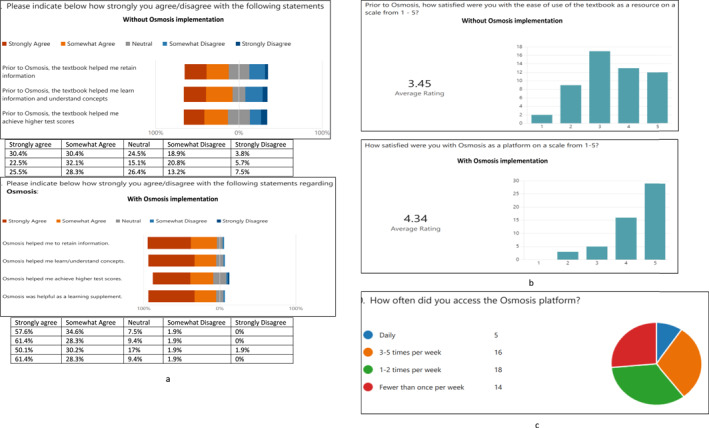
Results of pre and post treatment student experience survey (a, b). Analysis of student experience feedback data. Student experience survey insights and summary of qualitative open‐ended student feedback data showing (a) perceived effectiveness of Osmosis on various learning outcomes and retention, (b) user satisfaction level, and (c) frequency of use.

Fifty‐three out of 86 students in the treatment group completed the student experience survey after the completion of the semester. Students used a Likert scale to indicate their level of agreement or disagreement with statements about both the textbook and Osmosis's utility in helping them to retain information, learn, and understand new concepts, and achieve higher test scores. Figure 2a illustrates that students consistently rated Osmosis higher than the textbook in Likert scale responses. Students agreed that Osmosis helped them to retain information. Most students (92.2% ± 0.042%) either strongly agreed or somewhat agreed that Osmosis helped them retain information, while a smaller percentage (60.8% ± 0.086%) reported the same for the textbook. Similarly, 90.2% ± 0.045% of students agreed that Osmosis helped them learn and understand concepts, compared to 54.9% ± 0.086% for the textbook. The pattern continued for the percentage of students that agreed that Osmosis helped them achieve higher test scores compared to the textbook, with 80.4% ± 0.052% agreeing for Osmosis to be helpful, while 54.9% ± 0.086% for the textbook to be helpful.

The survey overwhelmingly found Osmosis to be a helpful learning supplement, with 90.2% ± 0.045% of students either strongly or somewhat agreeing to its usefulness (Figure 2a). Students expressed their positive feedback for the multimedia learning platform Osmosis through an average rating of 4.39 out of 5, which is considerably higher than that of the course textbook which received an average rating of 3.45 out of 5 (Figure 2b). Most students reported that they accessed Osmosis more than once per week, with the most common weekly access ranging from one to five times (Figure 2c).

These findings suggest that students generally perceived Osmosis as a more effective learning resource than the textbook. Osmosis was consistently rated higher across all three categories of information retention, concept learning, and test score improvement.

Students indicated which features of Osmosis they found most helpful as a learning tool, in an open answer survey question included in the questionnaire. We reviewed and categorized responses into the following categories: videos, quizzes, content/platform, notes/clinical correlates, flashcards, pictures, and other/NA. Responses show that students found the videos, quizzes, and the overall content/platform to be the most helpful features (Table 1). However, our student survey data showed that the videos were the most preferred feature of this multimedia platform.

**TABLE 1:** Summary of student responses about the most helpful feature of Osmosis as a learning resource.

Response category	Response count	Percent
Videos	21	41%
Quizzes	14	27%
Content/concepts/platform	14	27%
Notes	8	16%
Flashcards	6	12%
Pictures	4	8%
Other/NA	7	14%

*Note*: Comparative analysis of student feedback performed on open‐ended questions regarding user experience/preferences.

A list of the traditional and nontraditional learning resources other than the course textbook, used by the students to retain and learn/understand course‐related information prior to using Osmosis, was compiled from the responses to the open‐ended questions numbered 6 and 7 from the survey questionnaire, respectively (Table 2). Based on this data, the in‐class lectures and presentations by instructors as well as slides and lecture notes uploaded by them on the LMS were the most used learning resource by students for both retaining course‐related information and understanding concepts.

**TABLE 2:** Summary of student responses about the resources used other than the textbook to retain and learn course‐related information prior to receiving access to Osmosis platform.

Prior to Osmosis	Help to retain information		Help to learn information (understanding concepts)	
Resources other than textbook	Response count	Percent	Response count	Percent
Class/lectures/presentations by professors	23	36%	22	34%
Slides/powerPoints/PDF/lecture notes/class notes	19	30%	8	12%
Quizlets	5	8%	7	11%
Self‐study notes from other sources	6	10%	4	6%
Google/Internet searches	0	0%	10	16%
Class captures/recordings	7	11%	2	3%
YouTube videos	4	6%	11	17%

### 3.3 Correlation between student performance and usage of specific features

The usage metrics from the platform revealed that students watched a median of 34 videos per week, answered a median of 30 questions per week, and completed a median of 37 flashcards per week. During the study period, students watched 3520 videos, answered 8612 questions, and answered 31,022 flashcards (Table 3). Pearson's correlation coefficient was calculated to examine the relationship between the average final grade and various usage statistics (number of videos watched, number of questions answered, and number of flashcards answered), which showed some positive correlation for all of them.

**TABLE 3:** Analysis of Osmosis student usage metrics and their correlation with course performance.

Usage metric	Correlation with average final grades (*r* value)	Median weekly count	Average confidence indicated
Questions answered	0.279 (*p* = 0.010)	30	77%
Flashcards answered	0.151 (*p* = 0.169)	37	85%
Videos watched	0.140 (*p* = 0.202)	34	N/A

**FIGURE 3:**
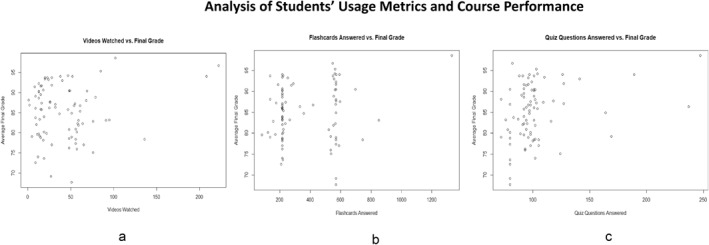
Correlation analysis between the students' final course grades and (a) number of videos watched, (b) number of flashcards attempted, and (c) number of quiz questions answered. Sample correlation coefficients calculated examined the relationship between the usage metrics and final grades in the two courses. There was a moderate positive correlation between the number of questions answered and final grades (*r* = 0.279, *p* < 0.05) as well as a weak positive correlation between the number of flashcards answered and final grades (*r* = 0.151, *p* = 0.169), as well as number of videos watched and final grades (*r* = 0.140, *p* = 0.202). These results suggest that the more students used the tool, the higher their grades tended to be.

The distribution of the students' average final grades against the number of videos watched, the number of questions answered, and number of flashcards answered also showed a similar trend. Sample correlation coefficients calculated and examined the relationship between the usage metrics and final grades in the two courses. Both Figure 3 and Table 3 shows a moderate positive correlation between the number of questions answered and final grades (*r* = 0.279, *p* < 0.05) as well as a weak positive correlation between the number of flashcards answered and final grades (*r* = 0.151, *p* = 0.169), as well as number of videos watched and final grades (*r* = 0.140, *p* = 0.202). These results suggest that the more students used the tool, the higher their grades tended to be.

Logistic regression analysis was performed between videos watched, embedded quiz questions answered, and flashcard questions answered, to examine the relationship between students' access to Osmosis and final exam scores and final course grades. Fitting a logistic regression model to the data revealed that students with access to the multimedia learning resource were more likely to score 90% or higher in the final exam in Pharmacology. The odds ratio was 2.88 for Pharmacology and 1.02 for Podiatric Medicine. On the other hand, analyzing the relationship between Osmosis access and a course grade of 90% or higher revealed odds ratio values of 2.71 for Podiatric Medicine and 1.30 for Pharmacology. Together, these results indicate that students having access to Osmosis in addition to traditional learning resources were more likely to score high on the final exam in Pharmacology and secure a high final grade in Podiatric Medicine.

## 4 DISCUSSION

Students tasked with learning challenging material can benefit greatly from research on the use of multimedia‐based digital learning platforms, such as Osmosis, to complement the traditional teaching and learning methods—whether the findings are implemented by educators or students find ways to incorporate them on their own. Numerous studies have explored the impact of implementing similar educational resources on learning outcomes. Research has shown that implementing an audiovisual module improved clinical exam outcomes for medical interns [14]. Audiovisual, story‐based mnemonics improved exam performance as well as time to recall in a randomized, controlled study of first year medical students [8]. In addition, a meta‐analysis on knowledge acquisition indicates that animations have a positive effect over static graphics [7].

Student preference can be a pivotal factor in determining the best way to deliver challenging material. Students who spend more time with study materials give themselves more opportunities to understand difficult concepts. A group of medical students given study materials incorporating video content indicated that they felt it was the clearest way to learn new concepts and held their attention better [15]. Online problem‐based learning materials that incorporated multimedia animation have been shown to improve undergraduate medical students' knowledge, skills, and attitude [16]. Implementing short videos and case studies into veterinary school curriculum increased student confidence; Students also reported that the short videos were useful and motivating [17]. Our survey data indicated that students found the Web‐based digital resource useful for learning concepts, retaining information, and were more satisfied with the usability of Osmosis as a learning tool compared to the textbook. This is consistent with previous research demonstrating increased student engagement, comprehension, and satisfaction with multimedia‐based resources compared to traditional instructional methods [18, 19].

Studies reported by Gilbert et al. showed that investigating and identifying the specific aspects of a learning tool which are effective in improving exam scores and course outcomes can help inform students' study habits and incorporation of spaced repetition with flashcards improved exam scores for first‐year medical students [20]. Medical students that used virtual reality videos to prepare for surgical procedures reported that they would like more videos used for this purpose [9]. Meta‐analysis found that video‐based education and 3D animations improve knowledge acquisition, reduce training time, learning duration, and improve surgical skill acquisition [21].

The results reported in our study revealed a positive correlation between the number of videos watched, the number of questions answered, and the number of flashcards answered with final grades for the treatment group. This suggests that students who used Osmosis more frequently were more likely to perform better in the final exams. However, it is important to note that correlation does not equal causation. The students chose the videos as their most preferred feature within the Osmosis platform, which is in alignment with the principles of CTML, since the videos can present visual, auditory, as well as cognitive processing channels simultaneously, making learning more effective [22]. In general, the students felt that using a multimedia‐based learning tool such as Osmosis helped them to learn concepts and retain information though their exam performance showed mixed results. However, it is important to remember that students participating in our study overwhelmingly felt that class lectures presented, and other learning materials uploaded by instructors helped them understand, learn, and retain information too. A case study on university level lecture taught in a large class size reported similar results [23]. So overall, the results of this study suggest that Osmosis is a promising multimedia learning tool for supporting student learning by complementing traditional content delivery in podiatric medical courses. However, more research is needed to confirm the effectiveness of Osmosis and to understand its long‐term impact on student learning outcomes.

In our quasi‐experiment comparing student exam scores, the participants were not randomized. This reduces the capability and aptness of drawing conclusions from any observed differences; in quasi‐experiments, it is difficult to determine if the intervention alone caused the difference in outcomes. The potential difference in student aptitude between the two groups is somewhat mitigated by the fact that both groups were second‐year medical students at the same college with similar average incoming GPA at the start of the course. The absence of pretest observations comparing the Treatment group to the Control group makes it difficult to determine whether differences between the two groups were due to the intervention or could be attributed to unmeasured confounding variables. The study was conducted at a single institution, which limits its generalizability. Therefore, it is possible that the results of the study would not be the same at other institutions. Third, the study relied on student self‐report data for some of its measures. Self‐reported data can be biased, so it is important to interpret any results cautiously. The mixed results of prior research and the difference in the results obtained from the same cohorts in the two studied courses suggest that the effectiveness of multimedia learning may depend on various factors, such as the specific multimedia tool used, the way it was implemented to provide spaced repetition, the subject matter being taught, and the characteristics of the learners. Therefore, factors such as the difficulty of subject matter and extent of contact with the concepts covered in the videos and frequency of usage should be considered when planning the integration of an audiovisual learning tool into the curriculum to enhance students' learning in order to ensure better outcomes.

The two most likely influential unmeasured confounding variables in our study are potential differences in the overall academic aptitude, which of the students in each sample [24], as well as potential differences in the amount of time spent with study materials between the two groups due to the spaced repetition provided in the Osmosis platform that could impact the results. The potential difference in student aptitude between the two groups should be somewhat mitigated by the fact that both groups were second‐year medical students at the same college when being tested. Additionally, the two groups had similar average standardized test scores and mean incoming GPAs at the beginning of the semester. The absence of pretest observations comparing the Treatment group to the Control group makes it difficult to know whether any differences between the two groups could potentially be attributed to preexisting factors rather than the multimedia learning tool alone [25].

Future research examining multimedia learning tools' impact on exam scores in medical education should use a randomized controlled trial design to minimize the risk of confounding variables. In addition, a larger study that implements this type of learning resource at a larger scale, and in more preclinical and clinical courses throughout the curriculum, is needed to further analyze its effectiveness in the podiatric medical curriculum. Additional research could also explore the long‐term effects of multimedia learning on medical education. It is important to know whether the benefits of multimedia learning persist after students have completed their medical education.

## 5 CONCLUSIONS

The findings suggest that implementing a multimedia learning tool such as Osmosis may be an effective way to improve student‐learning outcomes. Students found Osmosis to be a helpful learning resource. Students rated Osmosis higher than the textbook in terms of its ability to help them retain information, learn new information, understand concepts, and achieve higher test scores. Students who had access to Osmosis outperformed those who did not, on the final exams for both courses. Additionally, students in the treatment group were more likely to score 90% or higher on the final exam for Pharmacology.

A moderate positive linear relationship between the number of questions a student answered, and the student's average final grade suggests that students who answered more questions tended to get higher final grades. These results imply that implementing the multimedia learning tool Osmosis in a medical school course may have a positive impact on student learning outcomes, suggesting that multimedia learning tools can be used to support student learning in a Podiatric medical curriculum. The data reported here do not support replacing traditional instructional and learning methods with multimedia‐based learning resources, but rather contributes to the mounting evidence backing the potential of these audiovisual web‐based tools in further enhancing the students' learning experience in traditional course content delivery and ultimately improving student learning outcomes. The limitations in the study design and implementation raises the possibility that the overall improvement reported here may not be solely attributable to the integration of Osmosis and could be due to the confounding factors such as comprehension and retention skills or increased contact time with the course topics provided by the platform features. Therefore, more research is needed to confirm the effectiveness of specific multimedia learning tools and to understand their long‐term impact on student learning outcomes.

## AUTHOR CONTRIBUTIONS

**Garrik Hoyt**: Conceptualization; data curation; formal analysis; methodology; writing – original draft; writing – review and editing. **Samuel Adegboyega**: Methodology; data curation; validation; writing –review and editing. **Gus Constantouris**: Methodology; data curation; validation; writing – review and editing. **Paramita Basu**: Conceptualization; methodology; supervision; writing – review and editing.

## CONFLICT OF INTEREST STATEMENT

The authors declared no potential conflicts of interest with respect to the research, authorship, and/or publication of this article.

## ETHICS STATEMENT

This study was approved by New York College of Podiatric Medicine’s (NYCPM) Institutional Review Board (IRB) in May 2022. Informed consent was waived by the IRB since students agree to the use of unidentifiable education data for research purposes at registration.

## Supporting information

Supporting Information S1

Supporting Information S2

Supporting Information S3

## Data Availability

The data that support the findings of this study are available from the corresponding author upon reasonable request.
