# Supplementary material for: Study of the impact of introducing a multimedia learning tool in podiatric medical courses
Source: J Foot Ankle Res. 2024 Jun 29;17(3):e12018. doi: 10.1002/jfa2.12018 (PMC11633368; doi:10.1002/jfa2.12018)
Supplement: Supplementary file 1 — Supporting Information S1 [file JFA2-17-e12018-s002.docx]

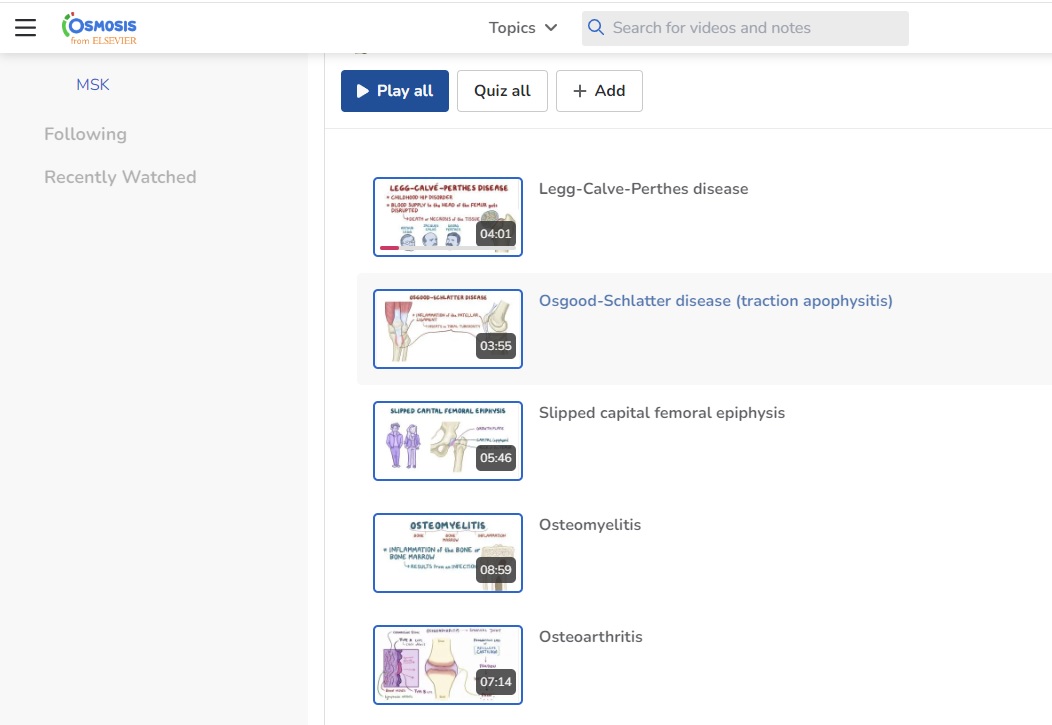

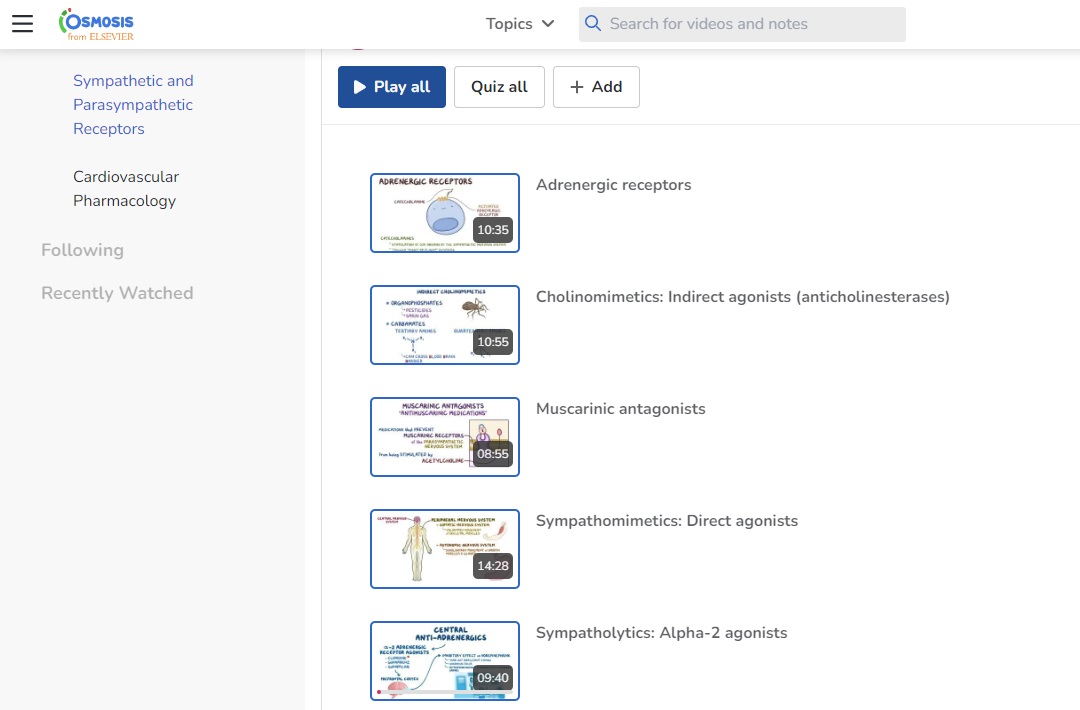


B

A

Appendix 1: Examples of instructor-assigned video playlists in the Osmosis digital learning platform in second year clinical and pre-clinical courses in (A) Podiatric medicine and (B) Pharmacology.
