# Supplementary material for: Study of the impact of introducing a multimedia learning tool in podiatric medical courses
Source: J Foot Ankle Res. 2024 Jun 29;17(3):e12018. doi: 10.1002/jfa2.12018 (PMC11633368; doi:10.1002/jfa2.12018)
Supplement: Supplementary file 2 — Supporting Information S2 [file JFA2-17-e12018-s003.docx]

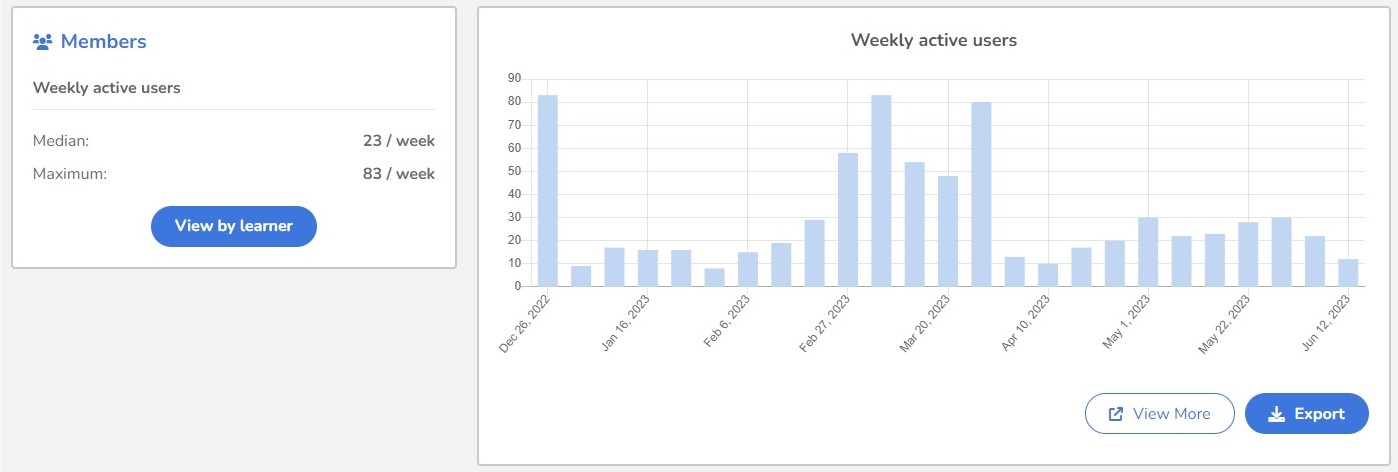


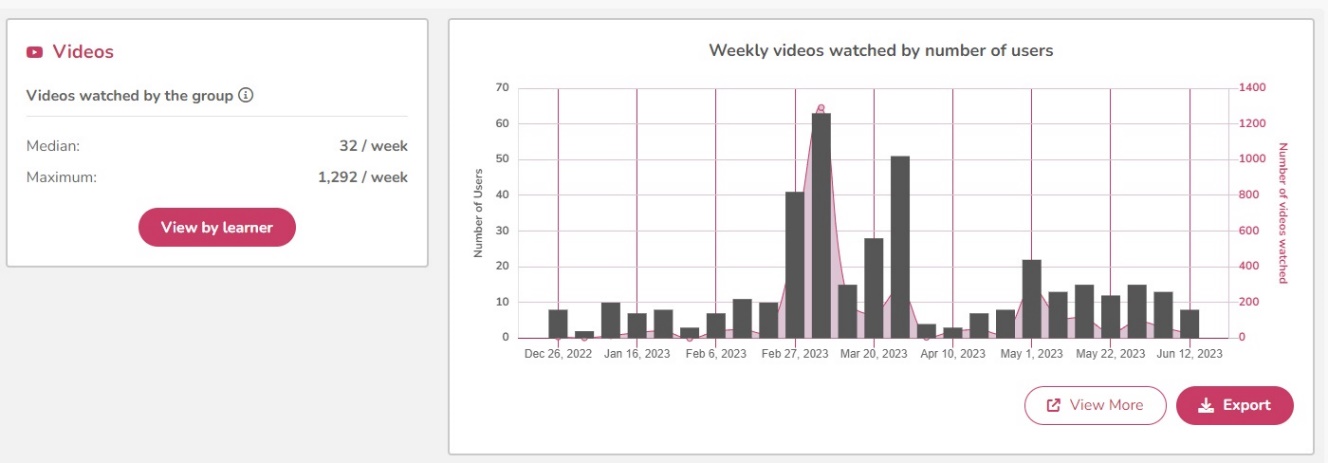


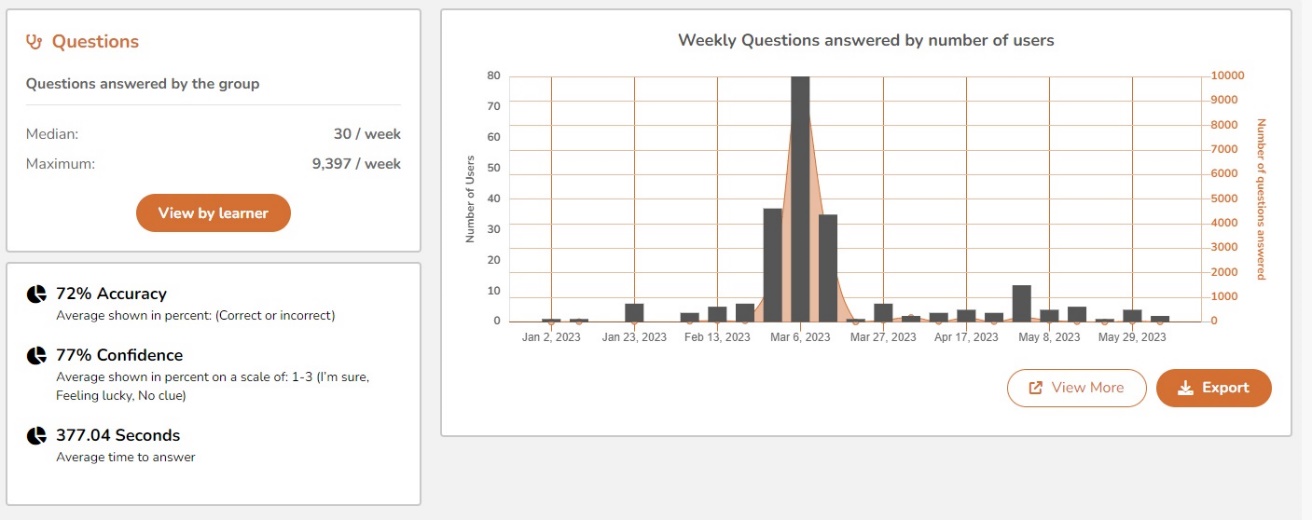


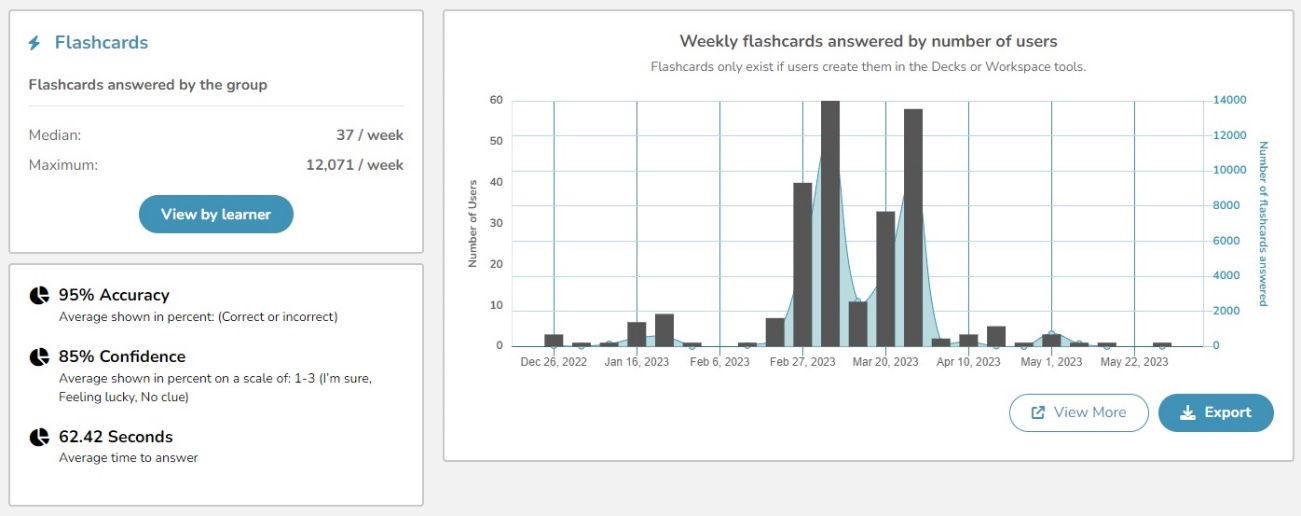


Appendix 2: A snapshot of platform usage metrics analytics for the instructor-assigned Osmosis videos and their associated questions and flashcards during the study period on the administrator’s dashboard
