# Supplementary material for: Study of the impact of introducing a multimedia learning tool in podiatric medical courses
Source: J Foot Ankle Res. 2024 Jun 29;17(3):e12018. doi: 10.1002/jfa2.12018 (PMC11633368; doi:10.1002/jfa2.12018)
Supplement: Supplementary file 3 — Supporting Information S3 [file JFA2-17-e12018-s001.pdf]

NYCPM Osmosis Student Survey

* Required

Baseline Questions

1. Please Indicate whether you received detailed information about the study including the objective and methodology used *

   ○ Yes

   ○ No

2. Please indicate which course are you referring to when filling out this survey *

   ○ Podiatric medicine

   ○ Pharmacology

3. Are you comfortable using digital technology and electronic resources for learning course related content? *

   ○ Yes

   ○ No

4. Do you have access to a computer or smart device and internet connection outside the college? *

   ○ Yes

   ○ No

Pre-Osmosis Questions

5. Please indicate below how strongly you agree/disagree with the following statements *

	Strongly Agree	Somewhat Agree	Neutral	Somewhat Disagree	Strongly Disagree
Prior to Osmosis, the textbook helped me retain information	◯	◯	◯	◯	◯
Prior to Osmosis, the textbook helped me learn information and understand concepts	◯	◯	◯	◯	◯
Prior to Osmosis, the textbook helped me achieve higher test scores	◯	◯	◯	◯	◯

6. Prior to Osmosis, what resources other than the textbook did you use to help you retain information ? *

7. Prior to Osmosis, what resources other than the textbook did you use to help you learn information (understanding concepts)? *

8. Prior to Osmosis, how satisfied were you with the ease of use of the textbook as a resource on a scale from 1 - 5? *

Post-Osmosis Questions

9. What specific features of Osmosis have been most helpful to you? *

10. Please indicate below how strongly you agree/disagree with the following statements regarding **Osmosis**: *

	Strongly Agree	Somewhat Agree	Neutral	Somewhat Disagree	Strongly Disagree
Osmosis helped me to retain information.	○	○	○	○	○
Osmosis helped me learn/understand concepts.	○	○	○	○	○
Osmosis helped me achieve higher test scores.	○	○	○	○	○
Osmosis was helpful as a learning supplement.	○	○	○	○	○

11. How often did you access the Osmosis platform? *

- Daily
- 3-5 times per week
- 1-2 times per week
- Fewer than once per week

12. How satisfied were you with Osmosis as a platform on a scale from 1-5? *
